# Homozygous Deletion of the Epigenetic Regulator *PHF20* in Individuals With Neurodevelopmental Disorder

**DOI:** 10.1155/humu/6484814

**Published:** 2025-12-22

**Authors:** Shira Yanovsky Dagan, Hongwen Xuan, Jonathan Rips, Emuna Paz-Ebstein, Talia Baer, Shira Gross, Ayala Frumkin, Xiaobing Shi, Tamar Harel

**Affiliations:** ^1^ Department of Genetics, Hadassah Medical Center, Jerusalem, Israel, hadassah.org.il; ^2^ Department of Epigenetics, Van Andel Institute, Grand Rapids, Michigan, USA, vai.org; ^3^ Faculty of Medicine, Hebrew University of Jerusalem, Jerusalem, Israel, huji.ac.il

**Keywords:** *Alu*–*Alu*-mediated deletion, exome sequencing, H4K16 acetylation, neurodevelopmental syndrome, PHF20

## Abstract

*PHF20* encodes plant homeodomain finger protein 20 (PHF20), a component of the KAT8‐containing nonspecific lethal (NSL) complex that deposits acetylation on histone H4 to activate gene expression. We report two unrelated individuals with developmental delay, microcephaly, and distinctive facial features, in whom exome sequencing and chromosomal microarray analysis revealed a homozygous deletion of *PHF20* that segregated with the disease phenotype in their families. Breakpoint junction sequencing revealed an *Alu–Alu*‐mediated deletion event. Western blot in cells from an affected individual showed undetectable PHF20, while levels of other NSL complex subunits were unaltered. Transcriptomic and epigenomic analysis revealed significant downregulation of gene pathways related to cell projection and neuronal development, associated with reduced histone H4K16 acetylation at these genes. In conclusion, our data suggest that homozygous deletion of *PHF20* leads to a neurodevelopmental syndrome, potentially through targeted epigenetic dysregulation and altered gene expression essential for neuronal development. Identifying additional families with biallelic *PHF20* variants will further delineate the phenotypic spectrum, and molecular studies in neuronal cell lines will be essential for understanding the disease mechanism.

## 1. Introduction

Epigenetic regulation allows for alternative gene expression in different tissues and includes histone modifications, DNA methylation, and chromatin remodeling. These modifications are coordinated by writers, erasers, readers, and remodelers. *PHF20* encodes plant homeodomain finger protein 20 (PHF20), an epigenetic reader of dimethylated lysine 4 of histone H3 (H3K4me2).

The two KAT8‐containing complexes, the male‐specific lethal (MSL) complex and the nonspecific lethal (NSL) complex, deposit acetylation on histone H4K16 and other residues on the H4 tail and also on nonhistone proteins [1, 2]. PHF20 is stably associated with the NSL complex, which partakes in maintaining cellular homeostasis and is linked to multiple cellular processes including transcriptional regulation, DNA damage response, autophagy control, and cell reprogramming [3–6]. Binding of PHF20 to H3K4me2 is required for NSL‐mediated histone acetylation and transcriptional activation of target genes. Thus, the PHF20/NSL complex “reads” methylation and “writes” acetylation, functionally coupling these epigenetic modifications [1–3].

PHF20 is required for the generation and reprogramming of embryonic stem cells (ESCs) and induced pluripotent stem cells (iPSCs). It controls key reprogramming and pluripotency factors such as *OCT4* and *SOX2* and is targeted for ubiquitination and degradation by KDM6B/JMJD3 [7, 8]. In addition, PHF20 regulates the transcription of genes required for kinetochore assembly and centriole duplication [9], as well as proper osteoblast differentiation [10].

*Phf20*‐null mice die shortly after birth, although the exact cause of death was not deciphered. Null mice were born at a birthweight of ~50% of wild‐type mice, with a frail skeleton, missing lumbar vertebrae, and fewer areas of calcified bone. The bone marrow cellularity was reduced in knockout mice, the spleen demonstrated disorganization, and thymocytes were decreased, suggesting an early defect in hematopoietic stem/progenitor cell differentiation or proliferation [1].

In this study, we report two seemingly unrelated individuals with developmental delay, microcephaly, and distinctive facial features, who harbored a homozygous deletion encompassing *PHF20*. We show that this deletion results from *Alu–Alu*‐mediated recombination, and loss of *PHF20* leads to epigenetic and transcriptional alterations that may account for disease abnormalities.

## 2. Materials and Methods

### 2.1. Ethics Statement

Families were consented for research studies by informed consent, according to Helsinki committee–approved Protocol 0306‐10‐HMO.

### 2.2. Chromosomal Microarray

Genomic DNA was extracted from whole blood and applied to the Affymetrix CytoScan 750 K array (Thermo Fisher Scientific, Santa Clara, California, United States) according to the manufacturer′s instructions. The platform has a resolution level of 50 kb (25 probes). The arrays were scanned, and data were analyzed on the Affymetrix Chromosome Analysis Suite 3.1 (Genome Build 37, GRCh37).

### 2.3. Exome Sequencing

Exonic sequences from DNA were enriched with the xGen Exome Research Panel IDT‐V2 combined with xGen Human mtDNA Research Panel v1.0. Sequences were generated on a NovaSeq6000 sequencing system (Illumina, San Diego, California, United States) as 150‐bp paired‐end runs. The FASTQs were uploaded onto the Geneyx Analysis platform [11]. Alignment and variant calling of SNVs, SV, and copy number variants (CNVs) were called using Illumina DRAGEN Bio‐IT. The resulting VCF files were comprehensively annotated on the Geneyx Analysis annotation engine and presented for analysis, filtering, and interpretation. The human genome assembly hg19 (GRCh37) was used as reference.

### 2.4. Whole Genome Sequencing (WGS)

Genomic DNA was extracted from peripheral blood, and NGS libraries were prepared with an Illumina polymerase chain reaction (PCR)–free TruSeq DNA Library Prep Kit. Sequences were generated on an Illumina NovaSeq 6000 sequencing platform as 150 bp paired‐end reads, targeted for low coverage in order to resolve the identified breakpoint junction. The FASTQs were uploaded to the Geneyx Analysis platform [11]. Alignment and variant calling were performed using Illumina DRAGEN Bio‐IT, with the human genome assembly hg19 (GRCh37) as reference.

### 2.5. Multiplex PCR and Segregation

DNA was isolated from whole blood of the affected individual and from unaffected family members of both families. To amplify the breakpoint junction, PCR was carried out using the following primers: PHF20_F1: 5 ^′^‐CGAGGTTGTGGAAACTTGGT‐3 ^′^ and PHF20_R1: 5 ^′^‐TGCCTCCCTGGAAAAAGATA‐3 ^′^, with an expected product size of 916 bp. The primers did not amplify the wild‐type allele since the product was too large. PCR using primers within the deleted region were used to define the wild‐type allele, using the following primers: PHF20_F2: 5 ^′^‐CATCCAGCCCTGAAATCTGT‐3 ^′^ and PHF20_R2: 5 ^′^‐CCAGGTTGGCAAGACAATTC‐3 ^′^, yielding a product size of 299 bp. The resultant fragments were separated by 3% (*w*/*v*) agarose gel electrophoresis and subject to Sanger sequencing (BigDye Terminator v1.1 Cycle Sequencing Kit, Thermo Fisher).

### 2.6. Cell Culture

Lymphoblastoid cell lines (LCLs) from EBV‐transformed patient lymphocytes were maintained in RPMI‐1640 (Biological Industries, Beit Haemek, Israel) supplemented with appropriate serum, 1% L‐glutamine, and 1% penicillin–streptomycin antibiotics (Biological Industries, Beit Haemek, Israel).

### 2.7. CRISPR/Cas9 Genome Editing

A guide RNA (gRNA) targeting Exon 17 of *PHF20* (5 ^′^‐GTTTAACCTGCTGACCCATG‐3 ^′^) was designed by the CHOP CHOP design tool [12] (URL: https://chopchop.cbu.uib.no/) and was annealed to a tracrRNA before transfection. 1uM Cas9 protein and 1uM gRNA:tracrRNA were mixed to form an RNP complex in serum‐free medium (Opti‐MEM I Reduced Serum Medium, Invitrogen). HEK293T cells were transfected with the aid of the Lipofectamine CRISPRMAX Cas9 Transfection Reagent (Invitrogen), according to a reverse transfection protocol. Monoclonal cell populations were obtained by single cell sorting using fluorescence‐activated cell sorting (FACS) analysis. Sanger sequencing was used to determine CRISPR/Cas9 cleavage patterns in individual clones.

### 2.8. Western Blot (WB)

Whole cell extracts were extracted from WT or *PHF20* mutant LCLs, and proteins were separated by SDS‐PAGE gel and transferred to PVDF membranes as previously described [13]. After blocking with 5% nonfat milk, primary antibodies (H4 pan‐ac, Active Motif, 39925; H4K16ac, Abcam, ab109463; H3, Abcam, ab1791; PHF20, CST, 3934S; KANSL3, Sigma, HPA035018‐25UL; MSL2, CST, 44006S; KAT8, Abcam, ab200660; Actin, Sigma, A1978) were applied for overnight incubation at 4°C, followed by secondary antibodies for 1 h at room temperature. Images were developed with ECL Prime Reagent (Sigma, GERPN2236) using films in a dark room.

### 2.9. RNA‐Seq

Total RNA was purified from cultured cells with the TRIzol reagent. RNA‐seq was undertaken on LCLs in triplicates and compared to two controls—an unrelated healthy control and a heterozygous parent. Library preparation was followed by sequencing using the nonstrand‐specific protocol with poly‐A selection of mRNA (Illumina TruSeq) used in the GTEx sequencing project. Sequencing was performed in‐house on an Illumina NovaSeq 6000 sequencer. Following initial quality control (QC) assessment, normalization and differential expression analysis were executed with the DESeq2 package [14]. The differentially expressed genes (DEGs) were defined as genes with consistent up‐ or downregulation compared with both parental and unrelated controls and filtered by log2FoldChange > 0.6 with adjusted *p* value < 0.1. PANTHER GO analysis was done by Gene Ontology (https://geneontology.org), and the results were visualized by SRplot [15] with Top 10 enriched terms in up or down DEGs (FDR < 0.05).

### 2.10. cDNA Analysis

Total RNA was isolated from LCLs of the affected individuals and control individuals and from CRISPR/Cas9 *PHF20-*null HEK293T cells by TRIzol reagent extraction. cDNA was reverse transcribed from 1 *μ*g RNA using the qScript cDNA Synthesis Kit (Quantabio) which included oligo (dT) and random primers. Amplicons were quantified by real‐time quantitative PCR (qPCR) using PerfeCTa SYBR Green FastMix ROX (Quantabio) in the QuantStudio 5 Real‐Time PCR System (Thermo Fisher). *CLK2*, *GUSB*, and *RPLPO* served for normalization for the LCLs and HEK293T models, respectively. The qPCR experiments were performed in technical triplicates on three independent replicates (different flasks, grown and harvested at separate time points). Primers are provided in Supporting Information 2: Table S1.

### 2.11. ChIP and ChIP‐Seq Analysis

ChIP was performed as previously described [16]. In brief, 20 million cells were cross‐linked with 1% formaldehyde for 10 min and quenched with 125 mM glycine. The isolated nuclei were sonicated for 700 s using Covaris E220 Evo. For ChIP, 0.5 *μ*L H4K16ac antibody (Millipore, Cat. 07‐329) was added to 10 *μ*g chromatin. After five washes, DNA was reverse cross‐linked and purified using a PCR purification kit (Qiagen, Cat. 28106). ChIP‐seq libraries were constructed using the KAPA Hyper Prep Kit (Roche) and sequenced by Illumina NovaSeq 6000. ChIP‐seq reads were mapped to the hg38 human genome by HISAT2 (v2.1.0) [17] with parameter ‐‐no‐spliced‐alignment ‐k 1 ‐X 1000. Average profiles and heatmaps on protein‐coding genes were generated by Danpos2 (v2.2.2) [18].

## 3. Results

### 3.1. Clinical Reports (Table 1)

**Table 1:** Clinical characteristics of individuals with homozygous ~170 kbp deletion encompassing *PHF20*.

**Proband**	**Family A**	**Family B**
Gender	F	M
Age at last assessment	3.3 years	9 years
Ethnicity	Arab Muslim	Arab Muslim
Abnormal prenatal findings	Persistent left superior vena cava, VSD, and bilateral marginal hydronephrosis	NA
Gestational age at delivery (weeks + days)	40 + 6	38
Birthweight (grams/percentile)	2792 (< 3^rd^)	3200 (47^th^)
Head circumference at birth (cm/percentile)	33 (23^rd^)	NA
Birth defects	Perimembranous VSD (surgically repaired at 16 months)	Coronal hypospadias
Global developmental delay	Yes	Yes (DQ < 50 at 2.1 years)
Dysmorphism	Microcephaly, micrognathia, upslanting palpebral fissures, arched eyebrows with medial sparing, a large nose, prominent cupped, and low‐set ears, a thin upper lip, and slight clinodactyly	Brachycephaly, epicanthal folds, and cupped ears
Microcephaly	Yes (secondary)	Borderline (HC at 5^th^ percentile)
Neurologic examination	Shuffling gait	Shuffling gait, increased tone in lower limbs, and brisk reflexes
Hearing impairment	No	Yes
Ophthalmologic evaluation	NA	Astigmatism
Brain MRI	Microcephaly, small frontal lobes, and enlarged CSF spaces, possibly indicating brain atrophy	Normal (at 7 years)
EEG	NA	Normal
Genetic work‐up	CMA (single exome sequencing)	CMA (trio exome sequencing)

Abbreviations: CMA, chromosomal microarray; CSF, cerebrospinal fluid; DQ, developmental quotient; F, female; HC, head circumference; M, male; NA, not available; VSD, ventricular septal defect.

#### 3.1.1. Family A (Figure 1)

Figure 1Homozygous *Alu–Alu*‐mediated deletion encompassing *PHF20* in two affected families. (a) Low‐pass genome sequencing indicating ~170 kb deletion. (b) Pedigrees and segregation indicating homozygous deletion (916 bp band, spanning deletion) in both probands. The 299 bp band represents the wild‐type allele and is absent in the probands. Schematic design of primers is included underneath genotypes. (c) Breakpoint junction sequencing. Asterisks indicate identical nucleotides. Middle line (labelled as jx) represents the affected individual and shows the recombination between the proximal *AluY* (upper; blue font) and the distal *AluSq2* (lower; orange font). (d) Schematic diagram indicating Alu*–*Alu‐mediated recombination leading to deletion encompassing the entire *PHF20* gene.

**Figure:**
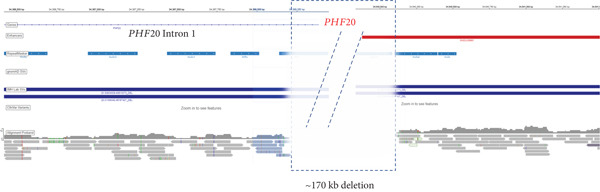
(a)

**Figure:**
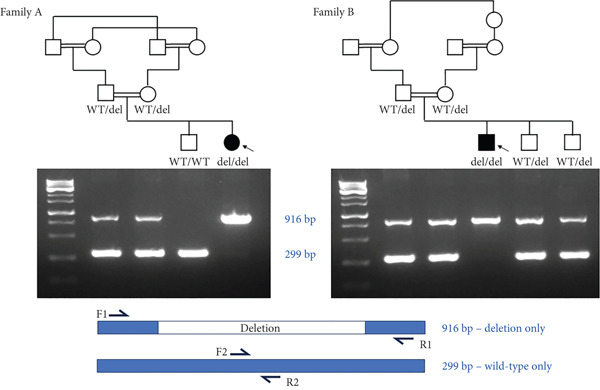
(b)

**Figure:**
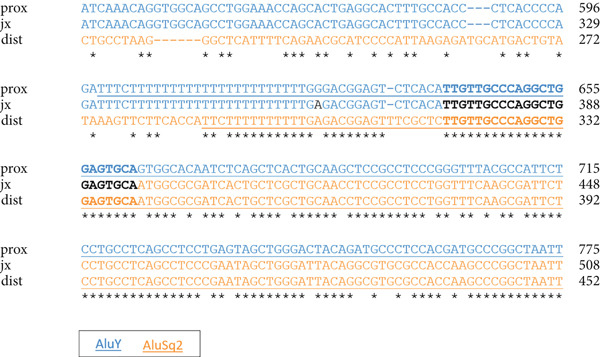
(c)

**Figure:**
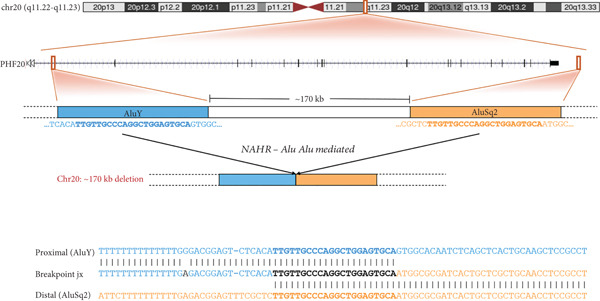
(d)

The proband was the second child of consanguineous parents of Arab Muslim origin. She was born at full term, following a pregnancy marked by abnormal prenatal findings including persistent left superior vena cava and marginal bilateral hydronephrosis of 5 mm. Fetal echocardiography identified a ventricular septal defect (VSD). She was delivered at 40 + 6 weeks of gestation, with a birth weight of 2792 g (< 3^rd^ percentile) and a head circumference of 33 cm (23^rd^ percentile). Postnatal echocardiography confirmed the presence of a perimembranous VSD, which was surgically repaired at 16 months of age. The child was referred to genetics due to developmental delay, primarily in speech, and the presence of a congenital heart defect. At 39 months, her physical examination revealed notable dysmorphic features including progressive microcephaly (head circumference 45.5 cm, < 3^rd^ percentile) (Supporting Information 1: Figure S1), micrognathia, upslanting palpebral fissures, arched eyebrows with medial sparing, a large nose, prominent cupped and low‐set ears, a thin upper lip, and slight clinodactyly. She also exhibited a shuffling gait. Brain MRI confirmed microcephaly, along with small frontal lobes and enlarged cerebrospinal fluid (CSF) spaces, possibly indicating brain atrophy (Supporting Information 1: Figure S2).

#### 3.1.2. Family B

The proband was the first child of consanguineous parents of Arab Muslim origin. He was delivered via urgent cesarean section at 38 weeks of gestation due to fetal distress. The pregnancy was otherwise unremarkable. His birth weight was 3200 g (47^th^ percentile). During his stay in the nursery, he failed the hearing screening and was later diagnosed with conductive hearing loss due to serous otitis media, necessitating multiple tympanostomy tube insertions. He presented with global developmental delay, beginning to walk at 20 months and speaking his first words at around 2.5 years. By the age of 6.5 years, his vocabulary remained significantly limited, with slurred pronunciation. A developmental assessment at 25 months of age revealed severe global delay, with a general developmental quotient (DQ) below 50. The child was first evaluated at a genetic clinic at 5.4 years of age. His physical examination revealed a head circumference of 48.5 cm, slightly below the third percentile, increased tone in the lower limbs with brisk reflexes, abnormal gait, and dysmorphic features including brachycephaly, epicanthal folds, and cupped ears. In addition, he had coronal hypospadias. Brain MRI at 7 years of age was normal. No clinical seizures were reported, and the electroencephalogram (EEG) was normal.

### 3.2. Homozygous ~170 kbp Deletion Encompassing *PHF20* Detected in Both Probands

Chromosomal microarray followed by exome sequencing was performed in the two unrelated patients and identified a rare homozygous ~170 kbp deletion at Chr20: 34,368,205–34,540,125 (hg19) (Figure 1a). The deletion included Exons 2–18 of *PHF20.* No single nucleotide variants of relevance to the phenotype were detected by exome sequencing. Multiplex PCR on genomic DNA derived from whole blood from the affected individuals and from unaffected family members validated this deletion and showed that it was present in a biallelic state only in the affected individuals (Figure 1b). It is noteworthy that there are no other homozygous loss‐of‐function events (CNVs or single nucleotide variants) in *PHF20* in publicly available databases nor in our local chromosomal microarray and next generation sequencing datasets. Submission of the gene into GeneMatcher [19] did not yield any biallelic events.

### 3.3. *PHF20* Deletion Was Mediated Through an *Alu*–*Alu* Recombination

To further investigate the genetic mechanism that caused that deletion, we undertook low‐pass WGS on an affected individual, followed by confirmation of the breakpoint junction by PCR and Sanger sequencing. This revealed a homozygous chimeric *Alu* element derived from an *AluY/AluSq2* recombination, with 22 bp microhomology at the apparent breakpoint junction (Figure 1c), suggesting that the deletion was caused by an *Alu–Alu* recombination (Figure 1d).

### 3.4. *PHF20* Deletion Results in Downregulation of Genes Important for Neuron Development

To determine how PHF20 loss leads to the phenotypic abnormalities, we established LCLs by EBV transformation of lymphocytes isolated from an affected individual and conducted RNA‐seq analysis. LCLs established from an unrelated unaffected individual and a heterozygous parent were used as controls. The intersection of DEGs compared to the controls identified 494 genes upregulated and 351 genes downregulated in the affected individual (Figure 2a and Supporting Information 2: Tables S2 and S3). PANTHER GO analysis of the DEGs revealed that while the upregulated genes were enriched in cytokine‐mediated signaling pathways and cellular response to cytokine (Figure 2b, upper panel), the downregulated genes were significantly enriched in cell projection and neuron development, which may account for the phenotypic abnormalities of the affected individual (Figure 2b, lower panel).

Figure 2Differentially expressed genes induced by PHF20 deletion. (a) Heatmap of *Z*‐score normalized up‐ and downregulated genes between controls and affected individuals. (b) PANTHER GO‐Slim Biological Process terms enriched from up or down DEGs. (c) Real‐time quantitative PCR (qPCR) results showing downregulation of selected genes in LCLs from affected versus control individuals. (d) qPCR results indicating downregulation of the same genes, in *PHF20*‐null HEK293T cells generated by CRISPR/Cas9 genome editing.

**Figure:**
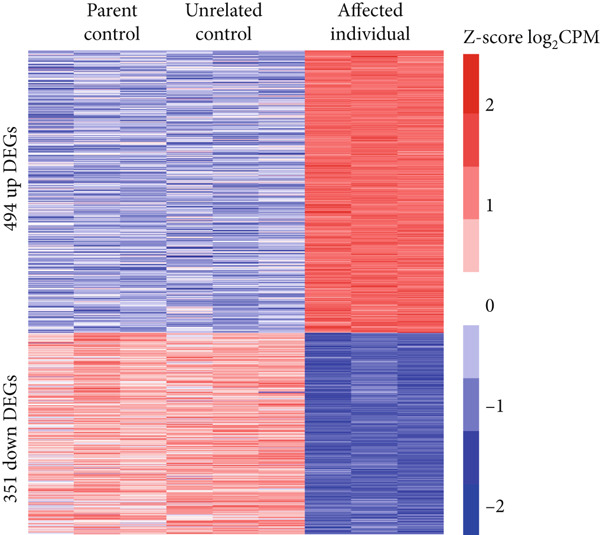
(a)

**Figure:**
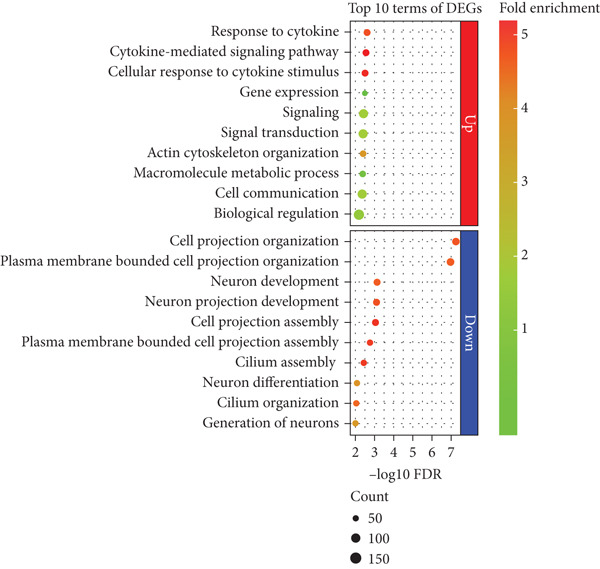
(b)

**Figure:**
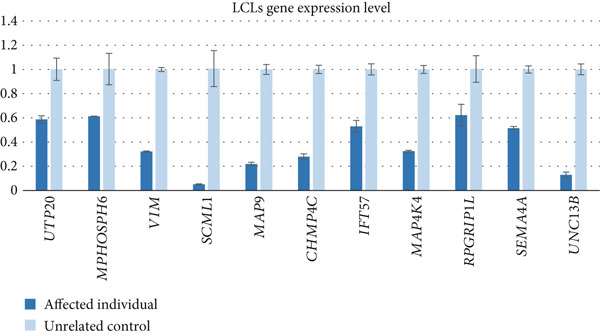
(c)

**Figure:**
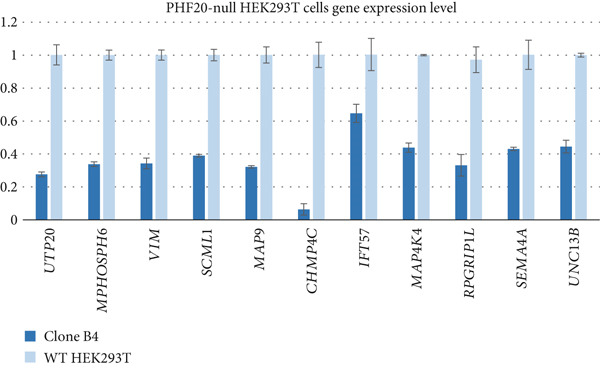
(d)

We further evaluated the differential expression of select genes within the downregulated pathways by qPCR in LCLs from an affected individual versus an unrelated control. We analyzed selected genes within the downregulated pathways, including genes involved in neuron development (*IFT57*, *MAP4K4*, *RPGRIP1L*, *SEMA4A*, and *UNC13B*). Significant differences in expression levels were confirmed for these genes (Figure 2c). Additionally, we investigated expression levels of specific genes (including those mentioned above and *UTP20*, *MPHOSPH6*, *VIM*, *SCML1*, *MAP9*, and *CHMP4C*) in CRISPR/Cas9‐edited *PHF20-*null HEK293T cells as an orthogonal model (Supporting Information 1: Figure S3) and confirmed downregulation of gene expression levels (Figure 2d), suggesting that the RNA‐seq data were robust. Expression levels in a heterozygous parent were intermediate between the affected child and unrelated control for several genes, while in others, the heterozygous parent was comparable to control (Supporting Information 1: Figure S4).

### 3.5. PHF20 Loss Alters Gene‐Specific H4K16ac Levels

PHF20 is stably associated with the KAT8‐containing NSL complex, which deposits acetylation on histone H4K16 and other residues on the H4 tail. We first determined whether the loss of PHF20 in cells impacts the integrity of the KAT8 associated complexes and histone acetylation. WB analysis of LCL cells from the affected individual and a healthy donor showed no detectable difference in KAT8, KAT8 complex components (KANSL3 and MSL2), or global H4K16ac levels, whereas as expected, PHF20 protein was undetectable in the affected individual (Figure 3a). These results suggest that PHF20 loss does not affect the integrity of KAT8 complexes and histone H4 acetylation globally.

Figure 3Genome‐wide H4K16ac distribution changes in LCL cells. (a) Western blot of KAT8 complexes and H4K16ac from control and affected individuals. (b) Average H4K16ac ChIP‐seq signals on gene bodies of all genes. (c) Heatmap of individual genes from (b). (d) Box plot of H4K16ac level on up or down DEGs in both samples. Unpaired two‐tailed Student′s *t*‐test was performed between control and affected individuals.

**Figure:**
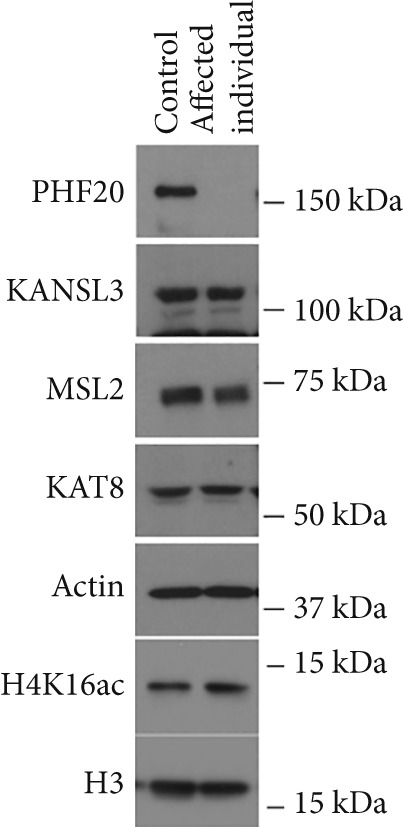
(a)

**Figure:**
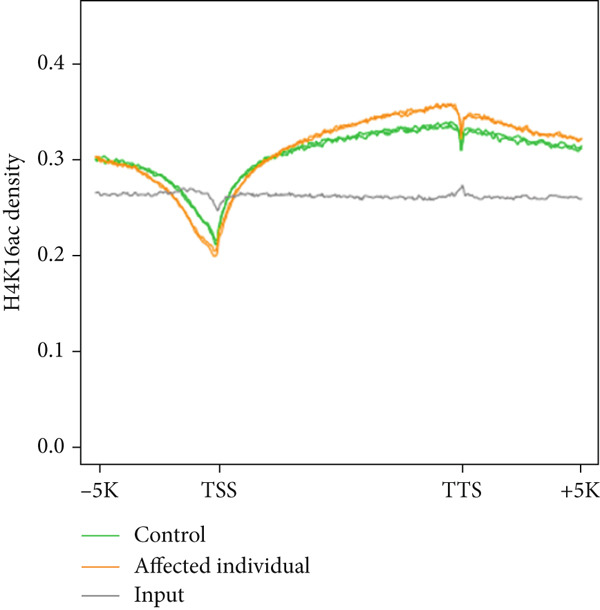
(b)

**Figure:**
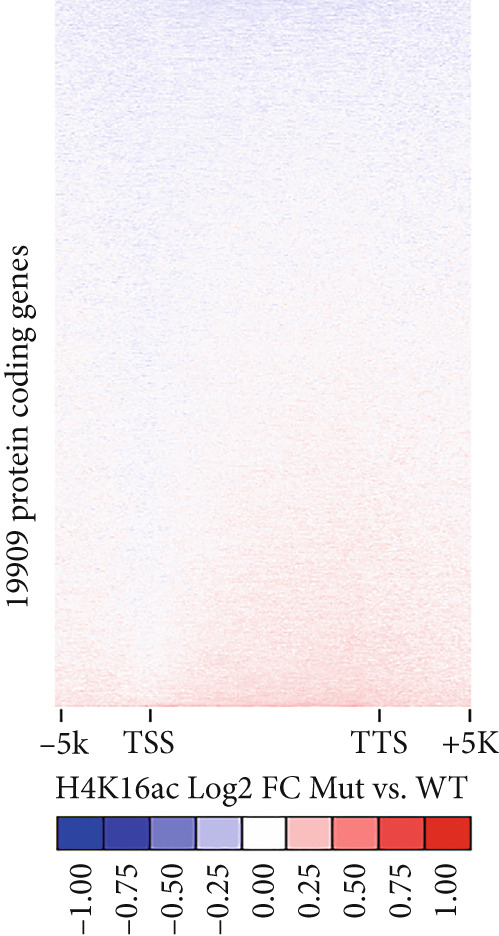
(c)

**Figure:**
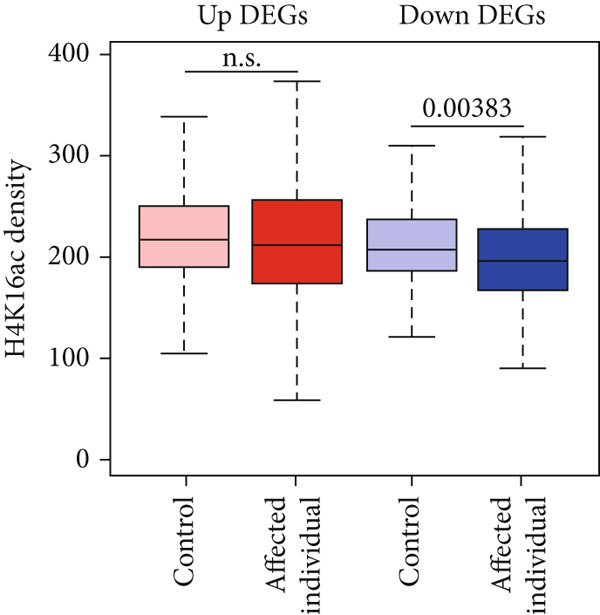
(d)

We reasoned that PHF20 loss might alter H4K16ac levels at specific genes or loci. To test this, we performed chromatin immunoprecipitation coupled with next generation sequencing (ChIP‐seq) for H4K16ac in the LCL cell lines established from the affected individual and the health donor. We found that the average profiles of H4K16ac ChIP‐seq densities were similar in both LCL cell lines, with a dip in the promoter region and a gradual increase in gene body toward the 3 ^′^ end of transcribed regions in the affected LCLs (Figure 3b). We ranked all protein‐coding genes by fold change of H4K16ac levels in the affected individual relative to the healthy control and found that indeed some genes showed reduced or increased H4K16ac in transcribed regions (Figure 3c). To assess whether these epigenetic changes impact gene expression, we evaluated H4K16ac levels in the up‐ and downregulated DEGs of both the control and affected individuals. We observed a significant decrease in H4K16ac levels in downregulated genes in the affected individual compared to the healthy control, while no significant changes were observed in the upregulated genes (Figure 3d). These results suggest that genes downregulated in the affected individual are likely direct targets of PHF20 and the NSL complex, whose dysregulation may impact neuron development.

## 4. Discussion

PHF20 partakes in transcriptional regulation, chromatin remodeling, and maintenance of cellular homeostasis [3, 6], yet its role in neurodevelopment has not yet been deciphered. In this study, we describe two seemingly unrelated affected individuals who were found to have an ~170 kbp homozygous deletion in *PHF20*, which segregated with the disorder within the respective families. Both individuals exhibited developmental delay, microcephaly, abnormal gait, and dysmorphic facial features. Notably, no hematological or immune issues were encountered, nor severe skeletal malformations, indicating that compensatory mechanisms may differ from those in the mouse null model [1].

The identified breakpoint junction mapped to a microhomology between directly oriented *Alu* sequences, suggesting that the deletion was mediated by an *Alu–Alu* recombination event. *Alu–Alu-*mediated rearrangements (AAMRs) are a form of microhomology‐mediated replicative repair involving template switching between homologous *Alu* elements [20, 21], and *Alu*‐associated CNVs have been estimated to cause at least ∼0.3% of human genetic diseases [20]. The first observed AAMR was described in 1987, in a patient with hypercholesterolemia and a 7.8 kbp deletion of *LDLR*[22]. Since then, numerous autosomal dominant diseases caused by AAMR have been identified, including Sotos syndrome (MIM 117550), Kabuki syndrome (MIM 147920), spastic paraplegia 4 (MIM 182601), and von Hippel–Lindau syndrome (MIM 193300) [20, 23]. Song et al. developed a machine learning–based tool, AluAluCNVpredictor, to predict AAMR events and highlighted human disease–associated genes in predicted AAMR hotspots [20]. Recently, a noncoding intronic deletion in *SLC16A2*, predicted to include transcription factor binding sites and a regulatory element, was detected by long‐read HiFi genome sequencing in a family with Allan–Herndon–Dudley syndrome (MIM 300523), underscoring the importance of long‐read sequencing for unsolved diagnostic odysseys [24]. Homozygous intragenic deletions mediated by *Alu–Alu* recombination and causing recessively inherited diseases have been reported in *BLM*, causing Bloom syndrome (MIM 210900), and in *SNX14*, causing spinocerebellar ataxia (MIM 616354) [25]. Homozygous full‐length gene deletions mediated by *Alu–Alu* recombination are rare but have also been described, as with *SLC13A5* in a family with epileptic encephalopathy [26].

Shared AOH around the homozygous deletion in *PHF20* suggested an ancestral event that was brought to homozygosity in both families by identity‐by‐descent. Interestingly, the heterozygous parents and other family members, some of whom are certainly heterozygous carriers, were unaffected. This is surprising given the constraint metrics, which would suggest haploinsufficiency–probability of loss‐of‐function intolerance (pLI) score = 1 and loss‐of‐function observed/expected upper bound fraction (LOEUF) score = 0.26 in gnomAD v2.1.1 [27].

Transcriptome analysis revealed the downregulation of pathways involved in cell projection and neuron development. Real‐time PCR confirmed the downregulation of genes such as *UNC13B*, *MAP4K4*, and *VIM*. Interestingly, although PHF20 loss does not affect KAT8 complex integrity and global histone H4 acetylation, we observed a specific reduction of histone H4K16 acetylation on genes involved in cell projection and neuron development. Importantly, decreased H4 acetylation is associated with the downregulation of gene expression in the affected individual, suggesting an epigenetic link between PHF20 loss and transcriptional outcomes.

This study adds to the growing number of genes encoding components of KAT8‐containing complexes and implicated in neurodevelopmental phenotypes. Pathogenic variants in *KAT8*, encoding lysine acetyltransferase‐8, lead to Li–Ghorgani–Weisz–Hubshman syndrome (MIM 618974), characterized by global developmental delay, seizures, dysmorphic features, and behavioral issues, with variable brain abnormalities [28]. Pathogenic variants or deletion of *KANSL1* underlie Koolen–De Vries syndrome (MIM 610443) which presents with developmental delay, intellectual impairment, and distinctive facial features [29]. Finally, pathogenic variants in *MSL2* lead to Karayol–Borroto–Haghshenas neurodevelopmental syndrome (MIM 620985), associated with developmental delay, intellectual disability, gait disturbance, dysmorphism, and autism. Interestingly, global H4K16ac levels were unchanged in iPSCs with *MSL2* variants, although expression of MSL2 targets was altered [30], similar to our findings for *PHF20*. The lack of a detectable difference in KAT8 complex components in our study suggests that PHF20 loss does not destabilize the complex, although PHF20 function is affected. Another possibility is that its homolog, PHF20L1, compensates for PHF20 in maintaining complex integrity. NSL complexes contain either PHF20 or PHF20L1, exclusively. Both PHF20 and PHF20L1 bind to promoters of highly expressed genes, yet a subset of target genes is bound by PHF20 only. Alternative compensatory mechanisms that sustain the transcript of critical genes have also been suggested, since deletion of either or both homologs does not abrogate gene expression of highly expressed genes or housekeeping genes [31]. Taken together, we suggest that while biallelic loss of PHF20 is not lethal due to partial compensation by either PHF20L1 or other mechanisms, it does lead to dysregulation of the transcription of selected genes.

A limitation of this study is the use of LCLs rather than neuronal cells. EBV transformation can alter genes and pathways in virus–host interactions, including cell cycle and mitosis [32]. We therefore sought to confirm our findings in an independent cellular model and generated *PHF20-*null HEK293T cells by CRISPR/Cas9 genome editing. The parental wild‐type cell line served as an isogenic control. Future studies in neuronal cells, brain organoids, or animal models will allow for a better understanding of the mechanism underlying *PHF20-*associated disorders in humans.

In conclusion, we present preliminary evidence that biallelic loss of *PHF20* leads to syndromic neurodevelopmental delay, possibly through dysregulation of acetylation and transcription of genes involved in cell projection and neuron development.

## Conflicts of Interest

The authors declare no conflicts of interest.

## Author Contributions

Shira Yanovsky Dagan and Hongwen Xuan contributed equally to this work.

## Funding

This study was funded by the Israel Science Foundation (10.13039/501100003977) (1149/24).

## Supporting Information

Additional supporting information can be found online in the Supporting Information section.

## Supporting information


**Supporting Information 1** Figure S1: Growth curves. Figure S2: Brain MRI (axial *T*2‐weighted) of Proband A at 14 months. Figure S3: CRISPR/Cas9 design and knockout clone. Figure S4: Expression levels of selected genes in affected individuals from RNA‐seq data.

**Supporting Information 2** Table S1: Primers used in the study. Table S2: Statistically significant downregulated genes compared to both controls. Table S3: Statistically significant upregulated genes compared to both controls.

## Data Availability

Data is available upon request from the authors.

## References

[1] Badeaux A. I. , Yang Y. , Cardenas K. , Vemulapalli V. , Chen K. , Kusewitt D. , Richie E. , Li W. , and Bedford M. T. , Loss of the Methyl Lysine Effector Protein PHF20 Impacts the Expression of Genes Regulated by the Lysine Acetyltransferase MOF, Journal of Biological Chemistry. (2012) 287, no. 1, 429–437, 10.1074/jbc.M111.271163, 2-s2.0-84855279809, 22072714. 22072714 PMC3249094

[2] Klein B. J. , Wang X. , Cui G. , Yuan C. , Botuyan M. V. , Lin K. , Lu Y. , Zhao Y. , Bruns C. J. , Mer G. , Shi X. , and Kutateladze T. G. , PHF20 Readers Link Methylation of Histone H3K4 and p53 With H4K16 Acetylation, Cell Reports. (2016) 17, no. 4, 1158–1170, 10.1016/j.celrep.2016.09.056, 2-s2.0-84994810198, 27760318. 27760318 PMC5125728

[3] Sheikh B. N. , Guhathakurta S. , and Akhtar A. , The Non-Specific Lethal (NSL) Complex at the Crossroads of Transcriptional Control and Cellular Homeostasis, EMBO Reports. (2019) 20, no. 7, e47630, 10.15252/embr.201847630, 2-s2.0-85066992051, 31267707. 31267707 PMC6607013

[4] Zhang X. , Peng D. , Xi Y. , Yuan C. , Sagum C. A. , Klein B. J. , Tanaka K. , Wen H. , Kutateladze T. G. , Li W. , Bedford M. T. , and Shi X. , G9a-Mediated Methylation of ER*α* Links the PHF20/MOF Histone Acetyltransferase Complex to Hormonal Gene Expression, Nature Communications. (2016) 7, no. 1, 10810, 10.1038/ncomms10810, 2-s2.0-84960510627, 26960573. PMC4792926 26960573

[5] Cui G. , Park S. , Badeaux A. I. , Kim D. , Lee J. , Thompson J. R. , Yan F. , Kaneko S. , Yuan Z. , Botuyan M. V. , Bedford M. T. , Cheng J. Q. , and Mer G. , PHF20 Is an Effector Protein of p53 Double Lysine Methylation That Stabilizes and Activates p53, Nature Structural & Molecular Biology. (2012) 19, no. 9, 916–924, 10.1038/nsmb.2353, 2-s2.0-84866091585, 22864287. PMC3454513 22864287

[6] Park S. W. , Kim J. , Oh S. , Lee J. , Cha J. , Lee H. S. , Kim K. I. , Park D. , and Baek S. H. , PHF20 Is Crucial for Epigenetic Control of Starvation-Induced Autophagy Through Enhancer Activation, Nucleic Acids Research. (2022) 50, no. 14, 7856–7872, 10.1093/nar/gkac584, 35821310. 35821310 PMC9371932

[7] Zhao W. , Li Q. , Ayers S. , Gu Y. , Shi Z. , Zhu Q. , Chen Y. , Wang H. Y. , and Wang R. F. , Jmjd3 Inhibits Reprogramming by Upregulating Expression of INK4a/Arf and Targeting PHF20 for Ubiquitination, Cell. (2013) 152, no. 5, 1037–1050, 10.1016/j.cell.2013.02.006, 2-s2.0-84874789264, 23452852. 23452852 PMC3742052

[8] Long W. , Zhao W. , Ning B. , Huang J. , Chu J. , Li L. , Ma Q. , Xing C. , Wang H. Y. , Liu Q. , and Wang R. F. , PHF20 Collaborates With PARP1 to Promote Stemness and Aggressiveness of Neuroblastoma Cells Through Activation of SOX2 and OCT4, Journal of Molecular Cell Biology. (2018) 10, no. 2, 147–160, 10.1093/jmcb/mjy007, 2-s2.0-85047087280, 29452418. 29452418 PMC5951121

[9] Pavlova G. A. , Popova J. V. , Andreyeva E. N. , Yarinich L. A. , Lebedev M. O. , Razuvaeva A. V. , Dubatolova T. D. , Oshchepkova A. L. , Pellacani C. , Somma M. P. , Pindyurin A. V. , and Gatti M. , RNAi-Mediated Depletion of the NSL Complex Subunits Leads to Abnormal Chromosome Segregation and Defective Centrosome Duplication in Drosophila Mitosis, PLoS Genetics. (2019) 15, no. 9, e1008371, 10.1371/journal.pgen.1008371, 2-s2.0-85072848578, 31527906. 31527906 PMC6772098

[10] Yang J. W. , Jeong B. C. , Park J. , and Koh J. T. , PHF20 Positively Regulates Osteoblast Differentiation via Increasing the Expression and Activation of Runx2 With Enrichment of H3K4me3, Scientific Reports. (2017) 7, no. 1, 10.1038/s41598-017-08868-0, 2-s2.0-85027521510, 28808306. PMC5556080 28808306

[11] Dahary D. , Golan Y. , Mazor Y. , Zelig O. , Barshir R. , Twik M. , Iny Stein T. , Rosner G. , Kariv R. , Chen F. , Zhang Q. , Shen Y. , Safran M. , Lancet D. , and Fishilevich S. , Genome Analysis and Knowledge-Driven Variant Interpretation With TGex, BMC Medical Genomics. (2019) 12, no. 1, 10.1186/s12920-019-0647-8, 31888639. PMC6937949 31888639

[12] Labun K. , Montague T. G. , Krause M. , Torres Cleuren Y. N. , Tjeldnes H. , and Valen E. , CHOPCHOP v3: Expanding the CRISPR Web Toolbox Beyond Genome Editing, Nucleic Acids Research. (2019) 47, no. W1, W171–W174, 10.1093/nar/gkz365, 2-s2.0-85069235112, 31106371. 31106371 PMC6602426

[13] Wan L. , Wen H. , Li Y. , Lyu J. , Xi Y. , Hoshii T. , Joseph J. K. , Wang X. , Loh Y. E. , Erb M. A. , Souza A. L. , Bradner J. E. , Shen L. , Li W. , Li H. , Allis C. D. , Armstrong S. A. , and Shi X. , ENL Links Histone Acetylation to Oncogenic Gene Expression in Acute Myeloid Leukaemia, Nature. (2017) 543, no. 7644, 265–269, 10.1038/nature21687, 2-s2.0-85015203567, 28241141. 28241141 PMC5372383

[14] Love M. I. , Huber W. , and Anders S. , Moderated Estimation of Fold Change and Dispersion for RNA-Seq Data With DESeq2, Genome Biology. (2014) 15, no. 12, 10.1186/s13059-014-0550-8, 2-s2.0-84924629414, 25516281. PMC4302049 25516281

[15] Tang D. , Chen M. , Huang X. , Zhang G. , Zeng L. , Zhang G. , Wu S. , and Wang Y. , SRplot: A Free Online Platform for Data Visualization and Graphing, PLoS One. (2023) 18, no. 11, e0294236, 10.1371/journal.pone.0294236, 37943830. 37943830 PMC10635526

[16] Xuan H. , Xu L. , Li K. , Xuan F. , Xu T. , Wen H. , and Shi X. , Hotspot Cancer Mutation Impairs KAT8-Mediated Nucleosomal Histone Acetylation, Journal of Molecular Biology. (2024) 436, no. 7, 168413, 10.1016/j.jmb.2023.168413, 38135180. 38135180 PMC10957314

[17] Kim D. , Paggi J. M. , Park C. , Bennett C. , and Salzberg S. L. , Graph-Based Genome Alignment and Genotyping With HISAT2 and HISAT-Genotype, Nature Biotechnology. (2019) 37, no. 8, 907–915, 10.1038/s41587-019-0201-4, 2-s2.0-85071193100, 31375807. PMC7605509 31375807

[18] Chen K. , Chen Z. , Wu D. , Zhang L. , Lin X. , Su J. , Rodriguez B. , Xi Y. , Xia Z. , Chen X. , Shi X. , Wang Q. , and Li W. , Broad H3K4me3 Is Associated With Increased Transcription Elongation and Enhancer Activity at Tumor-Suppressor Genes, Nature Genetics. (2015) 47, no. 10, 1149–1157, 10.1038/ng.3385, 2-s2.0-84942984698, 26301496. 26301496 PMC4780747

[19] Sobreira N. , Schiettecatte F. , Valle D. , and Hamosh A. , GeneMatcher: A Matching Tool for Connecting Investigators With an Interest in the Same Gene, Human Mutation. (2015) 36, no. 10, 928–930, 10.1002/humu.22844, 2-s2.0-84941877741, 26220891. 26220891 PMC4833888

[20] Song X. , Beck C. R. , Du R. , Campbell I. M. , Coban-Akdemir Z. , Gu S. , Breman A. M. , Stankiewicz P. , Ira G. , Shaw C. A. , and Lupski J. R. , Predicting Human Genes Susceptible to Genomic Instability Associated With *Alu*/*Alu*-Mediated Rearrangements, Genome Research. (2018) 28, no. 8, 1228–1242, 10.1101/gr.229401.117, 2-s2.0-85050885877, 29907612. 29907612 PMC6071635

[21] Mayle R. , Campbell I. M. , Beck C. R. , Yu Y. , Wilson M. , Shaw C. A. , Bjergbaek L. , Lupski J. R. , and Ira G. , DNA REPAIR. Mus81 and Converging Forks Limit the Mutagenicity of Replication Fork Breakage, Science. (2015) 349, no. 6249, 742–747, 10.1126/science.aaa8391, 2-s2.0-84940205642, 26273056. 26273056 PMC4782627

[22] Lehrman M. A. , Russell D. W. , Goldstein J. L. , and Brown M. S. , *Alu*-*Alu* Recombination Deletes Splice Acceptor Sites and Produces Secreted Low Density Lipoprotein Receptor in a Subject With Familial Hypercholesterolemia, Journal of Biological Chemistry. (1987) 262, no. 7, 3354–3361, 10.1016/S0021-9258(18)61510-8, 3818645. 3818645

[23] Shaw C. J. and Lupski J. R. , Implications of Human Genome Architecture for Rearrangement-Based Disorders: The Genomic Basis of Disease, Human Molecular Genetics. (2004) 13, no. supplement 1, R57–R64, 10.1093/hmg/ddh073, 14764619. 14764619

[24] Yoon J. G. , Lee S. , Park S. , Jang S. S. , Cho J. , Kim M. J. , Kim S. Y. , Kim W. J. , Lee J. S. , and Chae J. H. , Identification of a Novel Non-Coding Deletion in Allan-Herndon-Dudley Syndrome by Long-Read HiFi Genome Sequencing, BMC Medical Genomics. (2025) 18, no. 1, 10.1186/s12920-024-02058-4, 40033291. PMC11877835 40033291

[25] Mitani T. , Isikay S. , Gezdirici A. , Gulec E. Y. , Punetha J. , Fatih J. M. , Herman I. , Akay G. , Du H. , Calame D. G. , Ayaz A. , Tos T. , Yesil G. , Aydin H. , Geckinli B. , Elcioglu N. , Candan S. , Sezer O. , Erdem H. B. , Gul D. , Demiral E. , Elmas M. , Yesilbas O. , Kilic B. , Gungor S. , Ceylan A. C. , Bozdogan S. , Ozalp O. , Cicek S. , Aslan H. , Yalcintepe S. , Topcu V. , Bayram Y. , Grochowski C. M. , Jolly A. , Dawood M. , Duan R. , Jhangiani S. N. , Doddapaneni H. , Hu J. , Muzny D. M. , Marafi D. , Akdemir Z. C. , Karaca E. , Carvalho C. M. B. , Gibbs R. A. , Posey J. E. , Lupski J. R. , and Pehlivan D. , High Prevalence of Multilocus Pathogenic Variation in Neurodevelopmental Disorders in the Turkish Population, American Journal of Human Genetics. (2021) 108, no. 10, 1981–2005, 10.1016/j.ajhg.2021.08.009, 34582790. 34582790 PMC8546040

[26] Duan R. , Saadi N. W. , Grochowski C. M. , Bhadila G. , Faridoun A. , Mitani T. , Du H. , Fatih J. M. , Jhangiani S. N. , Akdemir Z. C. , Gibbs R. A. , Pehlivan D. , Posey J. E. , Marafi D. , and Lupski J. R. , A Novel Homozygous *SLC13A5* Whole-Gene Deletion Generated by Alu/Alu-Mediated Rearrangement in an Iraqi Family With Epileptic Encephalopathy, American Journal of Medical Genetics. Part A. (2021) 185, no. 7, 1972–1980, 10.1002/ajmg.a.62192, 33797191. 33797191 PMC8445493

[27] Karczewski K. J. , Francioli L. C. , Tiao G. , Cummings B. B. , Alföldi J. , Wang Q. , Collins R. L. , Laricchia K. M. , Ganna A. , Birnbaum D. P. , Gauthier L. D. , Brand H. , Solomonson M. , Watts N. A. , Rhodes D. , Singer-Berk M. , England E. M. , Seaby E. G. , Kosmicki J. A. , Walters R. K. , Tashman K. , Farjoun Y. , Banks E. , Poterba T. , Wang A. , Seed C. , Whiffin N. , Chong J. X. , Samocha K. E. , Pierce-Hoffman E. , Zappala Z. , O′Donnell-Luria A. H. , Minikel E. V. , Weisburd B. , Lek M. , Ware J. S. , Vittal C. , Armean I. M. , Bergelson L. , Cibulskis K. , Connolly K. M. , Covarrubias M. , Donnelly S. , Ferriera S. , Gabriel S. , Gentry J. , Gupta N. , Jeandet T. , Kaplan D. , Llanwarne C. , Munshi R. , Novod S. , Petrillo N. , Roazen D. , Ruano-Rubio V. , Saltzman A. , Schleicher M. , Soto J. , Tibbetts K. , Tolonen C. , Wade G. , Talkowski M. E. , Genome Aggregation Database Consortium , Neale B. M. , Daly M. J. , and DG M. A. , The Mutational Constraint Spectrum Quantified From Variation in 141,456 Humans, Nature. (2020) 581, no. 7809, 434–443, 10.1038/s41586-020-2308-7, 32461654. 32461654 PMC7334197

[28] Li L. , Ghorbani M. , Weisz-Hubshman M. , Rousseau J. , Thiffault I. , Schnur R. E. , Breen C. , Oegema R. , Weiss M. M. , Waisfisz Q. , Welner S. , Kingston H. , Hills J. A. , Boon E. M. , Basel-Salmon L. , Konen O. , Goldberg-Stern H. , Bazak L. , Tzur S. , Jin J. , Bi X. , Bruccoleri M. , K M. W. , Cho M. T. , Scarano M. , Schaefer G. B. , Brooks S. S. , Hughes S. S. , KLI G.van, Hagen J. M.van, Pandita T. K. , Agrawal P. B. , Campeau P. M. , and Yang X. J. , Lysine Acetyltransferase 8 Is Involved in Cerebral Development and Syndromic Intellectual Disability, Journal of Clinical Investigation. (2020) 130, no. 3, 1431–1445, 10.1172/JCI131145, 31794431. 31794431 PMC7269600

[29] Koolen D. A. , Kramer J. M. , Neveling K. , Nillesen W. M. , Moore-Barton H. L. , Elmslie F. V. , Toutain A. , Amiel J. , Malan V. , Tsai A. C. , Cheung S. W. , Gilissen C. , Verwiel E. T. , Martens S. , Feuth T. , Bongers E. M. , Vries P.de, Scheffer H. , Vissers L. E. , Brouwer A. P.de, Brunner H. G. , Veltman J. A. , Schenck A. , Yntema H. G. , and Vries B. B.de, Mutations in the Chromatin Modifier Gene KANSL1 Cause the 17q21.31 Microdeletion Syndrome, Nature Genetics. (2012) 44, no. 6, 639–641, 10.1038/ng.2262, 2-s2.0-84861587577, 22544363. 22544363

[30] Karayol R. , Borroto M. C. , Haghshenas S. , Namasivayam A. , Reilly J. , Levy M. A. , Relator R. , Kerkhof J. , McConkey H. , Shvedunova M. , Petersen A. K. , Magnussen K. , Zweier C. , Vasileiou G. , Reis A. , Savatt J. M. , Mulligan M. R. , Bicknell L. S. , Poke G. , Abu-El-Haija A. , Duis J. , Hannig V. , Srivastava S. , Barkoudah E. , Hauser N. S. , Born M.van den, Hamiel U. , Henig N. , Baris Feldman H. , McKee S. , Krapels I. P. C. , Lei Y. , Todorova A. , Yordanova R. , Atemin S. , Rogac M. , McConnell V. , Chassevent A. , Barañano K. W. , Shashi V. , Sullivan J. A. , Peron A. , Iascone M. , Canevini M. P. , Friedman J. , Reyes I. A. , Kierstein J. , Shen J. J. , Ahmed F. N. , Mao X. , Almoguera B. , Blanco-Kelly F. , Platzer K. , Treu A. B. , Quilichini J. , Bourgois A. , Chatron N. , Januel L. , Rougeot C. , Carere D. A. , Monaghan K. G. , Rousseau J. , Myers K. A. , Sadikovic B. , Akhtar A. , and Campeau P. M. , MSL2 Variants Lead to a Neurodevelopmental Syndrome With Lack of Coordination, Epilepsy, Specific Dysmorphisms, and a Distinct Episignature, American Journal of Human Genetics. (2024) 111, no. 7, 1330–1351, 10.1016/j.ajhg.2024.05.001, 38815585. 38815585 PMC11267526

[31] Van H. T. , Harkins P. R. , Patel A. , Jain A. K. , Lu Y. , Bedford M. T. , and Santos M. A. , Methyl-Lysine Readers PHF20 and PHF20L1 Define Two Distinct Gene Expression-Regulating NSL Complexes, Journal of Biological Chemistry. (2022) 298, no. 3, 101588, 10.1016/j.jbc.2022.101588, 35033534. 35033534 PMC8867114

[32] Dai Y. , Tang Y. , He F. , Zhang Y. , Cheng A. , Gan R. , and Wu Y. , Screening and Functional Analysis of Differentially Expressed Genes in EBV-Transformed Lymphoblasts, Virology Journal. (2012) 9, no. 1, 10.1186/1743-422X-9-77, 2-s2.0-84859069672, 22458412. PMC3433351 22458412

