# Supplementary material for: Homozygous Deletion of the Epigenetic Regulator PHF20 in Individuals With Neurodevelopmental Disorder
Source: Hum Mutat. 2025 Dec 22;2025:6484814. doi: 10.1155/humu/6484814 (PMC12721337; doi:10.1155/humu/6484814)
Supplement: Supplementary file 1 — Supporting Information 1 Figure S1: Growth curves. Figure S2: Brain MRI (axial T2‐weighted) of Proband A at 14 months. Figure S3: CRISPR/Cas9 design and knockout clone. Figure S4: Expression levels of selected genes in affected individuals from RNA‐seq data. [file HUMU-2025-6484814-s001.docx]

*
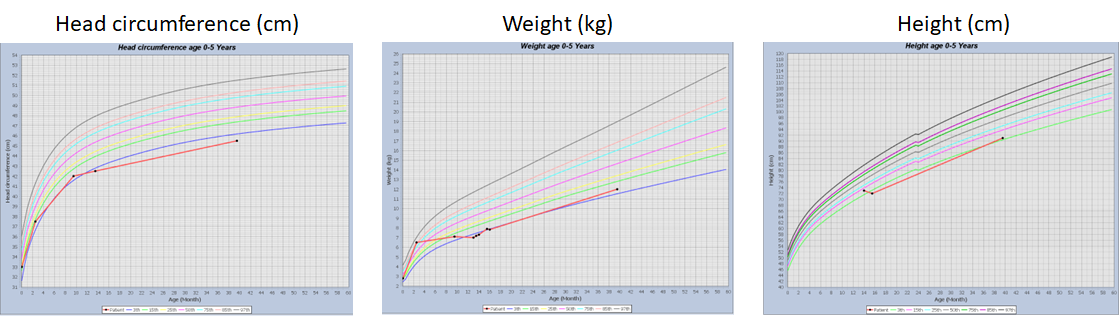
*

**Figure S1. Growth curves.** Head circumference, weight and height growth curves (0-5 years) in Proband A, indicating progressive microcephaly and borderline FTT and short stature.

**
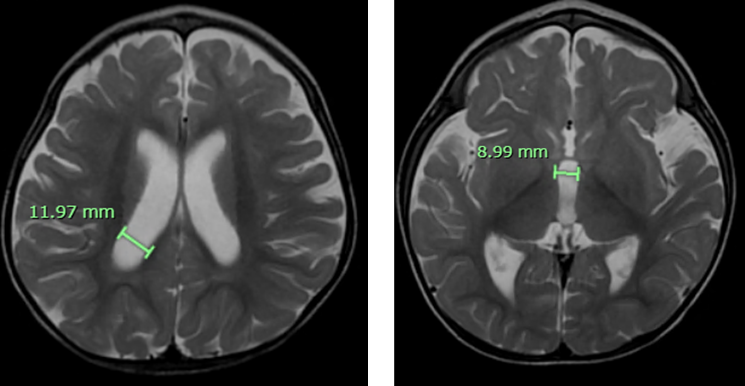
**

**Figure S2. Brain MRI (Axial T2-weighted) of Proband A at 14 months.** Mild bilateral dilatation of the lateral ventricles (up to 12 mm) and the third ventricle (up to 9 mm), consistent with mild ventriculomegaly.


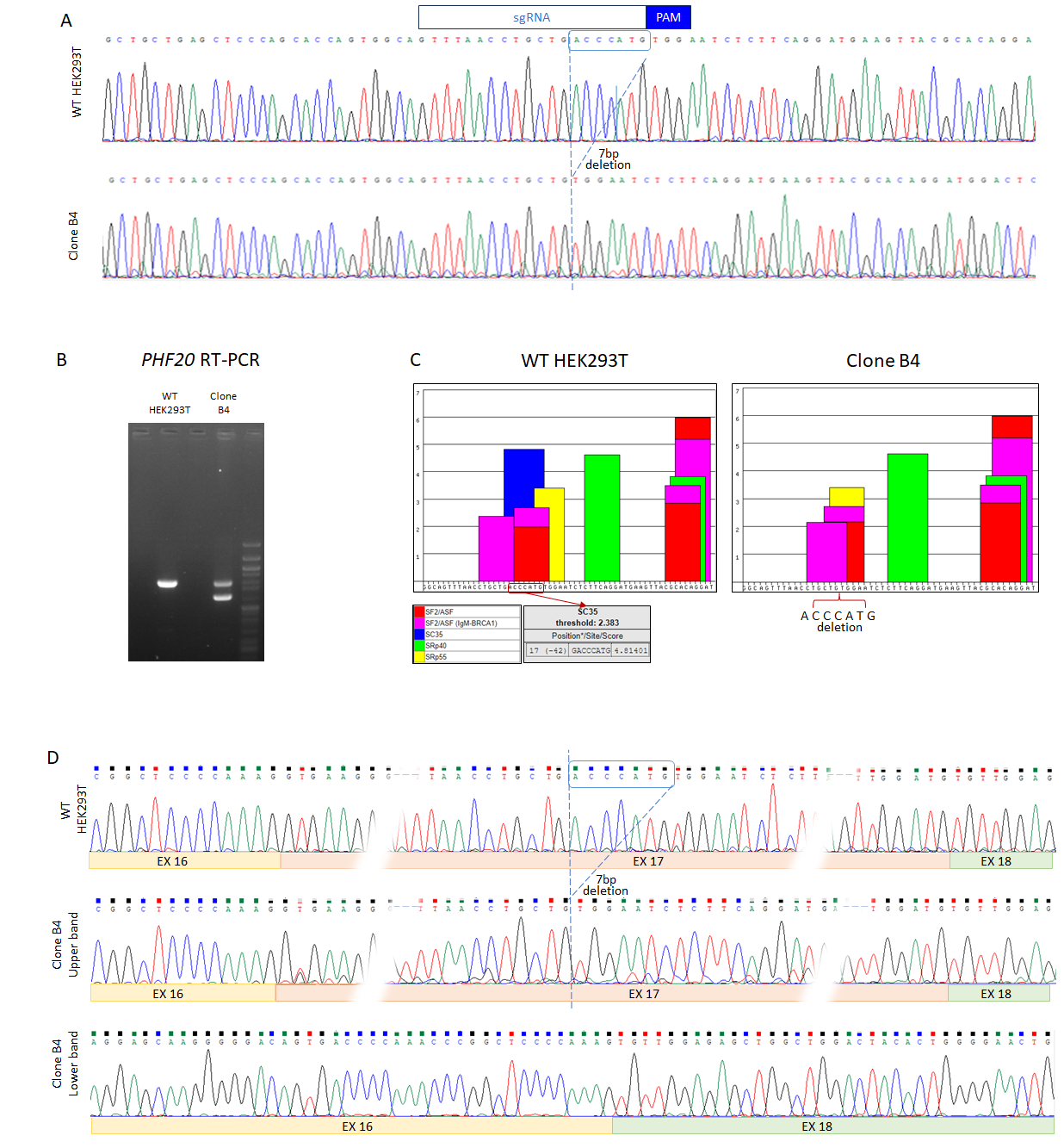


**Figure S3. CRISPR/Cas9 design and knockout clone.** (A) Wild-type sequence indicating gRNA and PAM sequence. Lower panel: Clone B4, with a 7bp deletion. (B) RNA analysis by RT-PCR and gel electrophoresis analysis shows smaller-sized band in Clone B4 corresponding to exon skipping. (C) ESEfinder software (<https://esefinder.ahc.umn.edu/cgi-bin/tools/ESE3/esefinder.cgi>) indicated that the 7bp deletion abolished a SC35 binding site. (D) Sanger sequencing indicating the wild-type HEK293T cells (upper panel), the 7bp deletion (middle panel) and exon skipping (lower panel).

**
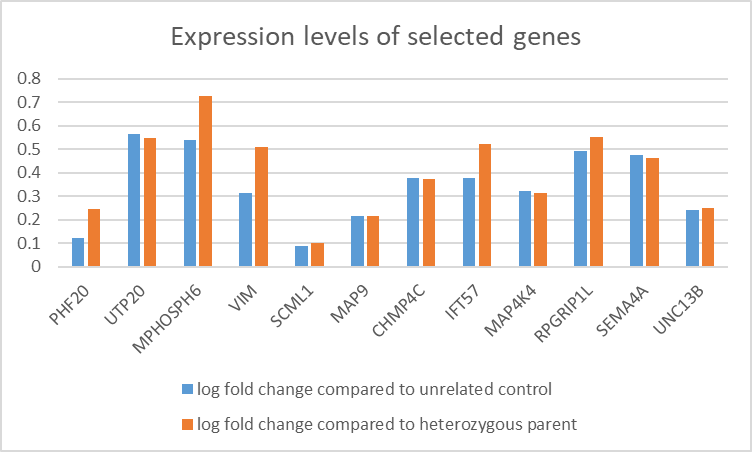
**

**Figure S4. Expression levels of selected genes in affected individual from RNA-seq data.** Data shown indicate log fold change of gene expression from RNA-seq experiments of affected LCLs, compared to unrelated control (blue) and healthy heterozygous parent (orange).
